# Supplementary material for: Quantification of fibroblast growth factor 23 and N-terminal pro-B-type natriuretic peptide to identify patients with atrial fibrillation using a high-throughput platform: A validation study
Source: PLoS Med. 2021 Feb 3;18(2):e1003405. doi: 10.1371/journal.pmed.1003405 (PMC7857735; doi:10.1371/journal.pmed.1003405)
Supplement: S1 Text — (DOCX) [file pmed.1003405.s002.docx]

**SUPPORTING INFORMATION (S1)**

Quantification of fibroblast growth factor 23 and N-terminal pro-B-type natriuretic peptide to identify patients with atrial fibrillation using a high-throughput platform: A validation study.

Winnie Chua^a^, Jonathan P. Law^a,b^, Victor R. Cardoso^a^, Yanish Purmah^a,b,c^, Georgiana Neculau^b,c^, Muhammad Jawad-Ul-Qamar^a,b,c^, Kalisha Russell^c^, Ashley Turner^c^, Samantha P. Tull^a^, Frantisek Nehaj^a,c^, Paul Brady^a,b,c^, Peter Kastner^d^, André Ziegler^e^, Georgios V. Gkoutos^f^, Davor Pavlovic^a^, Charles J. Ferro^a,b^, Paulus Kirchhof^a,b,c, g, h^, *Larissa Fabritz^a,b^.

^a^ Institute of Cardiovascular Sciences, University of Birmingham, Birmingham, United Kingdom.
^b^ University Hospitals Birmingham NHS Foundation Trust, Birmingham, United Kingdom.
^c^ Sandwell and West Birmingham Hospitals NHS Trust, Birmingham, United Kingdom.
^d^ Roche Diagnostics GmbH, Penzberg, Germany.
^e^ Roche Diagnostics International AG, Rotkreuz, Switzerland.
^f^ Institute of Cancer and Genomic Sciences, University of Birmingham, Birmingham, United Kingdom.

^g^ University Heart and Vascular Center UKE Hamburg, Hamburg, Germany.

^h^ German Center for Cardiovascular Research (DZHK), partner site Hamburg/Kiel/Lübeck, Germany.

**Table A in S1 Text:** **Re-estimated coefficients using data from the derivation cohort.**

| **Parameter** | **β coefficient** |
| --- | --- |
| **Age** (years) | 0.034 |
| **Sex** (male) | 0.517 |
| **BMI** | 0.053 |
| **FGF23** (Log_2_ scale) | 0.161 |
| **NT-proBNP** (Log_2_ scale) | 0.134 |
| Constant | -5.425 |

Data from the validation cohort were fitted with these coefficients.

**Table B in S1 Text: Comparison of model variables between the model development and the current validation cohort.**

|  | **Development** | | **Validation** | |
| --- | --- | --- | --- | --- |
|  | **No AF** (n = 215) | **AF** (n = 169) | **No AF** (585) | **AF** (n = 499) |
| **Age** (years) | 66 (57, 74) | 73 (63, 79) | 67 (57, 75) | 74 (65, 81) |
| **Sex** (Male) | 130 (61%) | 117 (69%) | 344 (59%) | 300 (60%) |
| **BMI** | 28.1 (25.2, 32.7) | 29.6 (26.0, 33.6) | 28.7 (25.0, 32.7) | 29.0 (25.1, 32.9) |
| **FGF23** (per 100 pg/mL), n = 198 | 1.57 (1.02, 2.39) | 1.92 (1.29, 4.22) | 1.55 (1.04, 2.62) | 1.93 (1.30, 4.16) |
| **NT-proBNP** (per 100 pg/mL), n = 150 | 3.09 (0.91, 12.51) | 7.91 (2.23, 20.95) | 4.25 (1.17, 15.70) | 12.00 (4.19, 30.15) |

Categorical variables are reported as n (%), continuous variables are reported as mean (standard deviation) or median (quartile 1, quartile 3) for non-parametric distributions (^*^). Data from both development and validation cohorts were obtained from the BBC-AF registry and recruited using the same criteria. Biomarker values reported are from the high-throughput pre-commercial assays. Note the different n numbers from those reported in the original development publication (Chua et al. 2019) as plasma were not available for all patients. FGF23, fibroblast growth factor 23; NT-proBNP, N-terminal pro-B-type natriuretic peptide.

**Table C in S1 Text: Descriptives of patients with biomarkers quantified using both Olink and Roche platforms by outcome group.**

| **Characteristic** | **No AF** | **AF** | **P-value** | **Univariable analysis** |
| --- | --- | --- | --- | --- |
|  | N = 321 | N = 272 |  | Odds ratio (95%CI) |
| **Age**, years * | 67 (58, 74) | 74 (65, 81) | <0.001 | 1.045 (1.030, 1.061) |
| **Sex,** males | 197 (61%) | 171 (63%) | 0.708 | 1.066 (0.764, 1.487) |
| **Ethnicity** |  |  | <0.001 |  |
| White | 218 (68%) | 238 (89%) | - | Reference |
| Asian | 66 (21%) | 18 (7%) | - | 0.250 (0.144, 0.434) |
| Black | 35 (11%) | 13 (5%) | - | 0.340 (0.175, 0.660) |
| **BMI,** kg/m^2^ * | 28.7 (25.5, 33.2) | 29.1 (25.0, 33.5) | 0.498 | 1.013 (0.987, 1.039) |
| **eGFR,** mL/min/1.73 m^2^ | 73.7 (22.9) | 66.4 (22.2) | <0.001 | 0.986 (0.979, 0.993) |
| **Diabetes** | 141 (44%) | 54 (20%) | <0.001 | 0.316 (0.218, 0.458) |
| **Stroke/TIA** | 36 (11%) | 28 (10%) | 0.719 | 0.908 (0.539, 1.532) |
| **Coronary artery disease** | 151 (47%) | 54 (20%) | <0.001 | 0.279 (0.193, 0.404) |
| **Hypertension** | 219 (68%) | 156 (57%) | 0.006 | 0.626 (0.448, 0.877) |
| **Heart failure** | 145 (45%) | 147 (54%) | 0.031 | 1.427 (1.032, 1.974) |
| **Admission criteria** (inpatient) | 264 (83%) | 179 (66%) | <0.001 | 0.408 (0.279, 0.598) |
| **Medication** |  |  |  |  |
| NOAC | 2 (1%) | 99 (36%) | <0.001 | 91.275 (22.239, 374.61) |
| VKA | 7 (2%) | 85 (31%) | <0.001 | 20.390 (9.238, 45.002) |
| Aspirin | 220 (67%) | 72 (27%) | <0.001 | 0.165 (0.116, 0.236) |
| Antiplatelet agents | 165 (51%) | 52 (19%) | <0.001 | 0.223 (0.154, 0.325) |
| ACE inhibitors | 98 (31%) | 69 (25%) | 0.164 | 0.773 (0.539, 1.111) |
| Angiotensin II receptor blocker | 57 (18%) | 49 (18%) | 0.935 | 1.018 (0.668, 1.551) |
| Beta-blocker | 187 (58%) | 143 (53%) | 0.165 | 0.794 (0.574, 1.100) |
| Diuretic | 106 (33%) | 123 (45%) | 0.002 | 1.674 (1.200, 2.337) |
| Calcium channel antagonist | 81 (25%) | 38 (14%) | 0.001 | 0.481 (0.314, 0.736) |
| Cardiac glycoside | 0 (0%) | 58 (21%) | - | - |
| Aldosterone antagonist | 17 (5%) | 21 (8%) | 0.230 | 1.496 (0.773, 2.897) |
| Antiarrhythmics | 6 (2%) | 29 (11%) | <0.001 | 6.265 (2.561, 15.331) |
| **Biomarkers** |  |  |  |  |
| FGF23 (per 100 pg/mL) * | 1.46 (0.97, 2.35) | 1.90 (1.23, 4.10) | <0.001 | 1.046 (1.023, 1.107) |
| NT-proBNP (per 100 pg/mL) * | 2.85 (0.95, 10.56) | 9.52 (2.78, 27.16) | <0.001 | 1.017 (1.010, 1.024) |
| FGF23 (per 100 pg/mL, Log_2_) * | 0.55 (-0.04, 1.23) | 0.92 (0.30, 2.03) | <0.001 | 1.437 (1.254, 1.647) |
| NT-proBNP (per 100 pg/mL, Log_2_) | 0.12 (2.33) | 1.63 (2.29) | <0.001 | 1.320 (1.225, 1.423) |

Categorical variables are reported as n (%), continuous variables are reported as mean (standard deviation) or median (quartile 1, quartile 3) for non-parametric distributions (^*^). The independent t-test (or Mann-Whitney U test for non-parametric distributions) and Χ^2^ tests were used to compare characteristics between patients. Comparing models in the same patients, the model calculated using Roche quantified biomarkers (AUC 0.689, 95%CI 0.647, 0.732) performed comparably to the model using Olink quantified biomarkers (AUC 0.680, 95%CI 0.637, 0.723). ACE, angiotensin-converting enzyme; BMI, body mass index; eGFR, estimated glomerular filtration rate by CKD-EPI formula; FGF23, fibroblast growth factor 23; NOAC, non-vitamin K antagonist oral anticoagulant; NT-proBNP, N-terminal pro-B-type natriuretic peptide; TIA, transient ischemic attack; VKA, vitamin K antagonist.

**Table D in S1 Text:** **Biomarker levels for patients with atrial flutter**.

| **Parameter** | **Atrial flutter** | **Sinus rhythm** | **P-value** |
| --- | --- | --- | --- |
| **FGF23** (per 100 pg/mL, Log^2^) | 0.75 (0.55, 1.69) | 0.64 (0.06, 1.39) | <0.001 |
| **NT-proBNP** (per 100 pg/mL, Log^2^) | 1.63 (2.51) | 0.56 (2.49) | 0.025 |
| **Parameter** | **Atrial flutter** | **Sinus rhythm** | **P-value** |
| **FGF23** (per 100 pg/mL, Log^2^) | 0.75 (0.55, 1.69) | 0.64 (0.06, 1.39) | <0.001 |
| **NT-proBNP** (per 100 pg/mL, Log^2^) | 1.63 (2.51) | 0.56 (2.49) | 0.025 |

| **Parameter** | **Atrial flutter** | **Sinus rhythm** | **P-value** |
| --- | --- | --- | --- |
| **FGF23** (per 100 pg/mL, Log^2^) | 0.75 (0.55, 1.69) | 0.64 (0.06, 1.39) | <0.001 |
| **NT-proBNP** (per 100 pg/mL, Log^2^) | 1.63 (2.51) | 0.56 (2.49) | 0.025 |

Median (Q1, Q3) is reported for FGF23 and mean (standard deviation, SD) for NT-proBNP. Comparison between patients with atrial flutter (n = 29) and patients in sinus rhythm were completed using the Mann-Whitney U (FGF23) and T-test (NT-proBNP) as appropriate. FGF23, fibroblast growth factor 23; NT-proBNP, N-terminal pro-B-type natriuretic peptide.

**Table E in S1 Text: Evaluation of NT-proBNP cutoffs by AF and heart failure statuses.**

|  | **NT-proBNP** | | | |
| --- | --- | --- | --- | --- |
|  | **<125 pg/mL** | **125 – 151 pg/mL** | **152 – 268 pg/mL** | **≥ 269 pg/mL** |
| **No AF, No HF** | 151 | 0 | 0 | 0 |
| **No AF, Yes HF** | 0 | 17 | 71 | 346 |
| **Yes AF, No HF** | 46 | 0 | 0 | 1 |
| **Yes AF, Yes HF** | 0 | 9 | 37 | 406 |
| **TOTAL** | 197 | 26 | 108 | 753 |

Counts of patients with NT-proBNP values within the three ranges (recommended rule-out cutoff <125 pg/mL, 5^th^ percentile 151 pg/mL, 10^th^ percentile 269 pg/mL) by AF and heart failure statuses. The recommended cutoff correctly rules out heart failure in patients with AF whereas higher cutoffs tend to misclassify patients. AF, atrial fibrillation; HF, heart failure; NT-proBNP, N-terminal pro-B-type natriuretic peptide.
