# Supplementary material for: Quantification of fibroblast growth factor 23 and N-terminal pro-B-type natriuretic peptide to identify patients with atrial fibrillation using a high-throughput platform: A validation study
Source: PLoS Med. 2021 Feb 3;18(2):e1003405. doi: 10.1371/journal.pmed.1003405 (PMC7857735; doi:10.1371/journal.pmed.1003405)
Supplement: S2 Text — (DOCX) [file pmed.1003405.s003.docx]

**SUPPORTING INFORMATION (S2)**

Quantification of fibroblast growth factor 23 and N-terminal pro-B-type natriuretic peptide to identify patients with atrial fibrillation using a high-throughput platform: A validation study.

Winnie Chua^a^, Jonathan P. Law^a,b^, Victor R. Cardoso^a^, Yanish Purmah^a,b,c^, Georgiana Neculau^b,c^, Muhammad Jawad-Ul-Qamar^a,b,c^, Kalisha Russell^c^, Ashley Turner^c^, Samantha P. Tull^a^, Frantisek Nehaj^a,c^, Paul Brady^a,b,c^, Peter Kastner^d^, André Ziegler^e^, Georgios V. Gkoutos^f^, Davor Pavlovic^a^, Charles J. Ferro^a,b^, Paulus Kirchhof^a,b,c, g, h^, *Larissa Fabritz^a,b^.

^a^ Institute of Cardiovascular Sciences, University of Birmingham, Birmingham, United Kingdom.
^b^ University Hospitals Birmingham NHS Foundation Trust, Birmingham, United Kingdom.
^c^ Sandwell and West Birmingham Hospitals NHS Trust, Birmingham, United Kingdom.
^d^ Roche Diagnostics GmbH, Penzberg, Germany.
^e^ Roche Diagnostics International AG, Rotkreuz, Switzerland.
^f^ Institute of Cancer and Genomic Sciences, University of Birmingham, Birmingham, United Kingdom.

^g^ University Heart and Vascular Center UKE Hamburg, Hamburg, Germany.

^h^ German Center for Cardiovascular Research (DZHK), partner site Hamburg/Kiel/Lübeck, Germany.

**Fig A in S2 Text: Violin plots for biomarkers.** NT-proBNP and FGF23 levels (in Log_2_ per 100pg/mL) by groups of patients with and without atrial fibrillation depicted using violin plots.


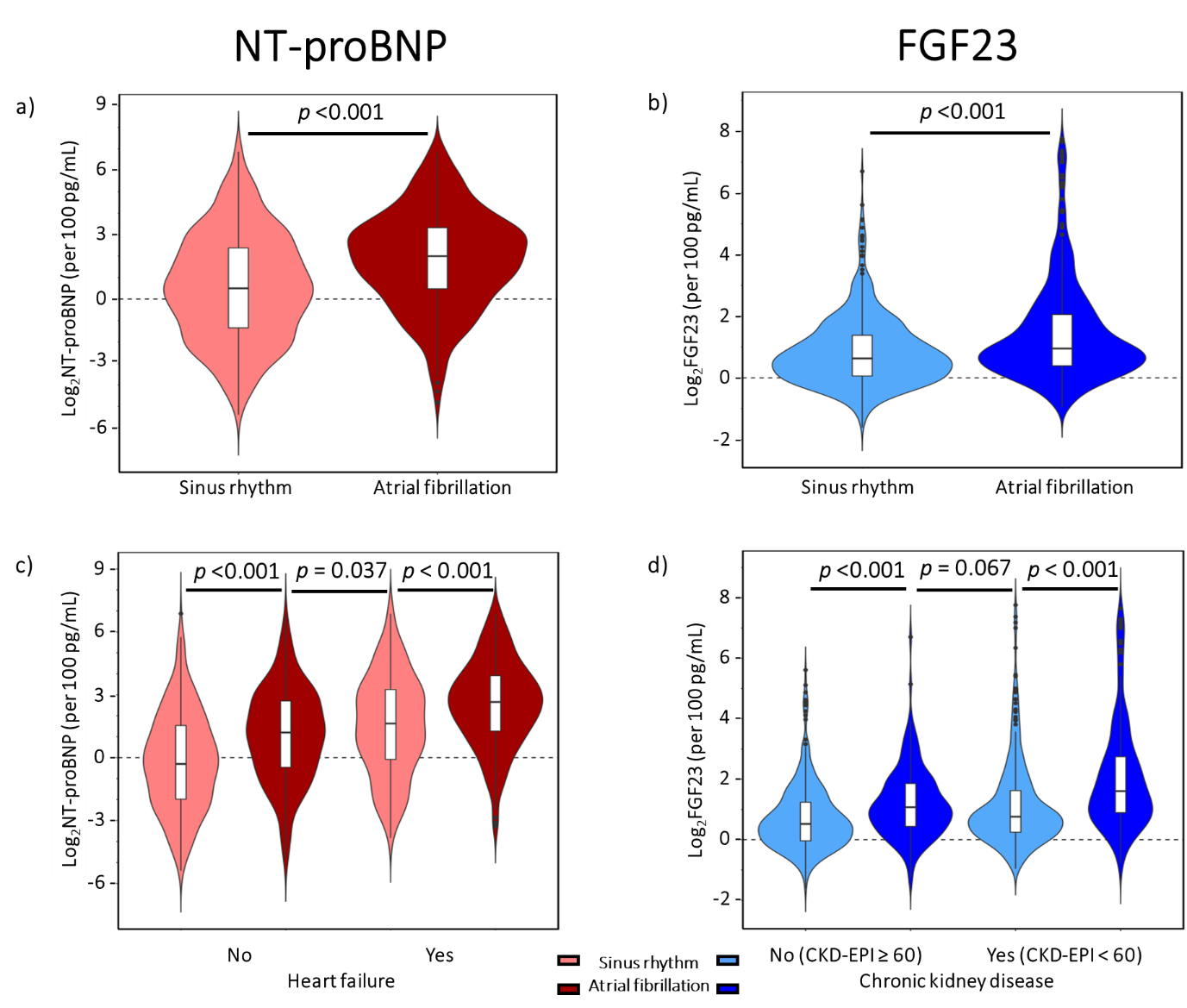


**Fig B in S2 Text: Adjustment of biomarkers to confounders.** Two adjustments were made for NT-proBNP and FGF23 with details as follows – Adjustment 1: age, sex, BMI, heart failure status; Adjustment 2: heart failure status, renal function calculated using the CKD-EPI equation; Adjustment 3: renal function calculated using the CKD-EPI equation, and Adjustment 4: renal function calculated using the CKD-EPI equation, heart failure status. Note that where the CKD-EPI is used, age, sex, and BMI are not included as confounders because the CKD-EPI equation already considers age and sex, normalised by body surface area.


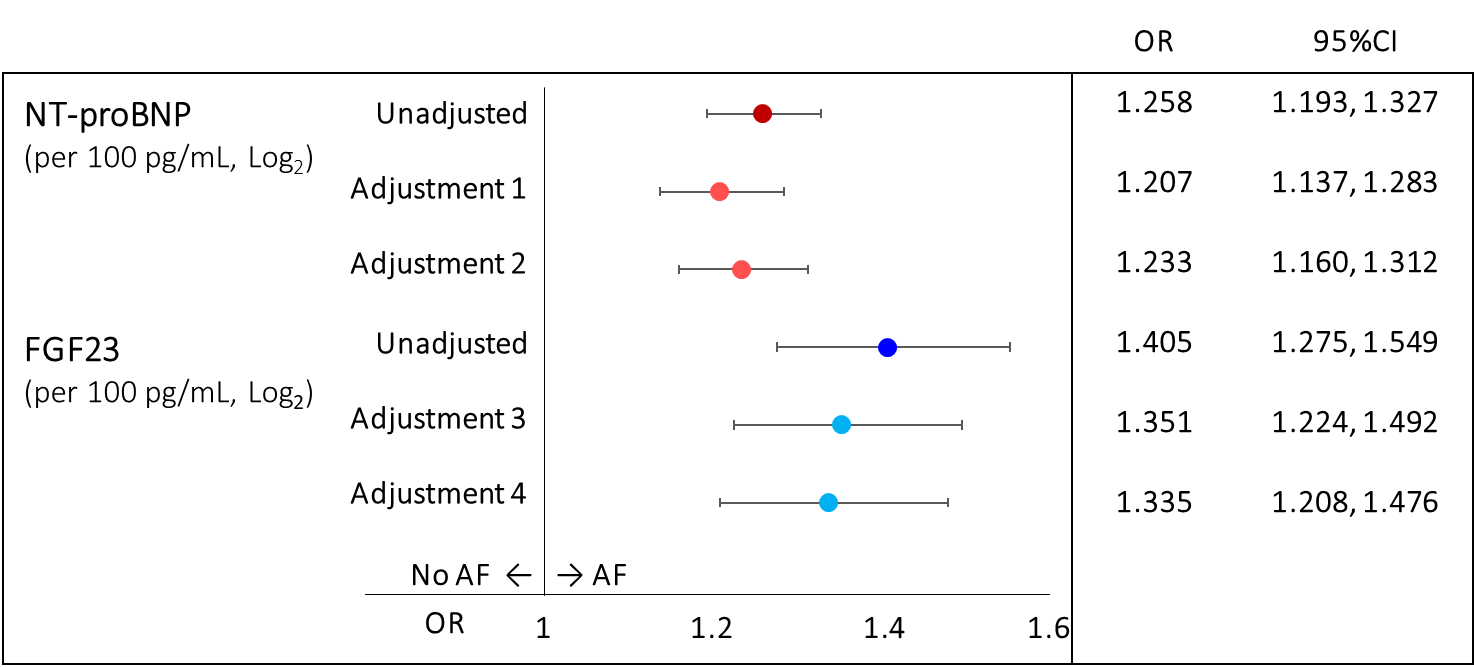


**Fig C in S2 Text: Discrimination slopes.** Visualisation of predictions between patients with and without atrial fibrillation using boxplots for the simple CHARGE-AF model and the biomarker model. The discrimination slope is calculated as the difference in mean predictions between the AF and no AF groups. For comparison, the discrimination slope for the CHARGE-AF model has been reported in the following cohorts: Pooled ARIC, CHS, and FHS = 0.056, AGES = 0.026, RS = 0.022[1].

CHARGE-AF, Cohorts for Heart and Aging Research in Genomic Epidemiology Atrial Fibrillation; ARIC, Atherosclerosis Risk in Communities; CHS, Cardiovascular Health Study; FHS, Framingham Heart Study; AGES, Age, Gene, and Environment Reykjavik Study; RS, Rotterdam Study.

**
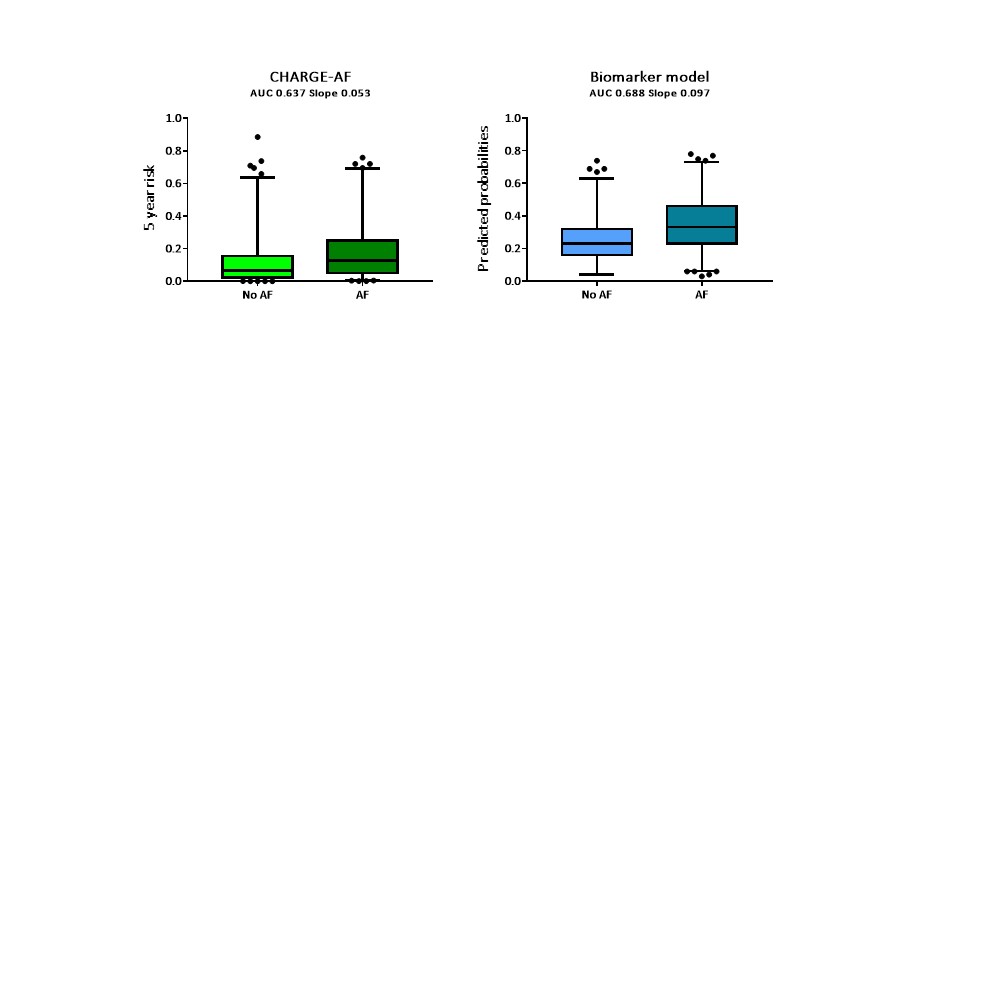
**

**Fig D in S2 Text:** **Sensitivity analysis for renal function.** FGF23 levels (in Log_2_ per 100pg/mL) by groups of patients with and without AF, by subcohorts above and below the mean/median of a) MDRD, b) CKD-EPI, and c) Cockcroft-Gault renal function equations. Patients with AF have consistently higher FGF23 levels than sinus rhythm patients. There is no difference in FGF23 levels between sinus rhythm patients with abnormal renal function and AF patients with normal renal function.


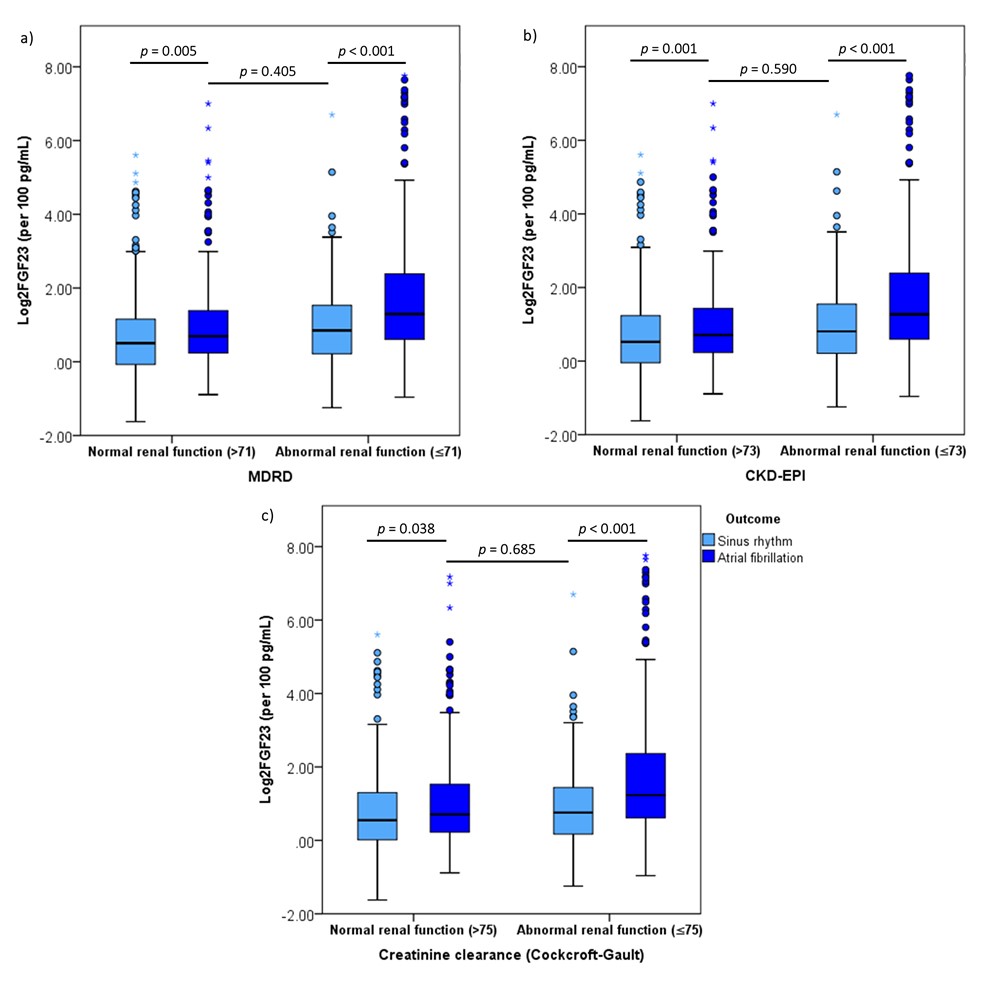


**REFERENCE**

1. Alonso A, Krijthe BP, Aspelund T, Stepas KA, Pencina MJ, Moser CB, et al. Simple risk model predicts incidence of atrial fibrillation in a racially and geographically diverse population: the CHARGE-AF consortium. J Am Heart Assoc. 2013;2(2):e000102. Epub 2013/03/30. doi: 10.1161/JAHA.112.000102. PubMed PMID: 23537808; PubMed Central PMCID: PMCPMC3647274.
