# Supplementary material for: Quantification of fibroblast growth factor 23 and N-terminal pro-B-type natriuretic peptide to identify patients with atrial fibrillation using a high-throughput platform: A validation study
Source: PLoS Med. 2021 Feb 3;18(2):e1003405. doi: 10.1371/journal.pmed.1003405 (PMC7857735; doi:10.1371/journal.pmed.1003405)
Supplement: Attachment — Submitted filename: reply to reviewers PLoS MEdicine R1 8June20LFwcLFnoTC.docx [file pmed.1003405.s004.docx]

**Reply to Requests from the editors:**

*Please revise your title according to PLOS Medicine's style. Your title must be nondeclarative and not a question. It should begin with the main concept/study question if possible followed by the study design in the subtitle after a colon (here, I assumed the data came from a prospective cohort but ": prognostic study" or ": prognostic validation study" could also be used).

Thank you, we have done that. The new title is:

*Quantification of fibroblast growth factor 23 and N-terminal pro-B-type natriuretic peptide to identify patients with atrial fibrillation using a high-throughput platform: validation study.*

*At this stage, we ask that you include a short, non-technical Author Summary of your research to make findings accessible to a wide audience that includes both scientists and non-scientists. The Author Summary should immediately follow the Abstract in your revised manuscript. This text is subject to editorial change and should be distinct from the scientific abstract. Please see our author guidelines for more information: <https://journals.plos.org/plosmedicine/s/revising-your-manuscript#loc-author-summary>

Thank you, we have included an Author Summary after the Abstract (*page 4*).

*In the last sentence of the Abstract Methods and Findings section, please also note briefly any key limitation(s) of the study's methodology.

Thank you, we have added this sentence at the end of the Abstract Methods and Findings section:

*The key limitation is that this validation was performed in a cohort which is very similar to the one used in model development, calling for further external validation.*

*It would be good to clarify in the Methods section whether the analytical approach used in the paper corresponded to that set out a priori in a prospective protocol or analysis plan? Please state this (either way) early in the Methods section.

We have included that this was a prospectively planned validation of our initial findings using a multimarker, semi-quantitative research assay (Olink).

a) If a prospective analysis plan (from your funding proposal, IRB or other ethics committee submission, study protocol, or other planning document written before analyzing the data) was used in designing the study, please include the relevant prospectively written document with your revised manuscript as a Supporting Information file to be published alongside your study, and cite it in the Methods section. A legend for this file should be included at the end of your manuscript.

Thank you. We planned this analysis prospectively in discussions with Roche Diagnostics (Peter Kastner and their team) after we had the initial results of our Olink discovery study (reference 5 of the current paper, Chua et al 2019). This is documented in several meeting protocols of our weekly lab meetings. Roche, who were an associate partner in the CATCH ME H2020 project, kindly agreed to accelerate the development of their FGF23 assay which is described in our paper. The plan as captured in those meeting minutes was to quantify BNP and FGF23 in the entire BBF-AF cohort to validate the model derived in the initial, Olink-based study. The rationale was to provide a biomarker quantification tool that can be implemented in clinical routine in the setting of routine lab parameters. We do not have a written analysis plan.

b) If no such document exists, please make sure that the Methods section transparently describes when analyses were planned, and when/why any data-driven changes to analyses took place.

To address the points above regarding an analysis plan, we have revised the Methods section accordingly. We have added the text and citations below:

*Following the initial discovery of FGF23 and NT-proBNP as markers for AF from a pool of 92 cardiovascular biomarkers(5), this analysis was prospectively planned as part of the extended work plan in the CATCH ME EU-funded project (6) to validate the findings using a high-throughput assay platform that can be implemented into routine clinical care. While the analyses were prospectively planned and agreed between the authors, we did not write a formal statistical analysis plan.*

c) In either case, changes in the analysis-- including those made in response to peer review comments-- should be identified as such in the Methods section of the paper, with rationale.

We have identified any additional analyses appropriately in the manuscript. In response to peer review comments, we have added the text below in the Methods section:

*In an additional sensitivity analysis, we also calculated the simple CHARGE-AF model(8, 9) for patients in whom all elements of the 11 variable score were available (n = 1050) and compared its performance to the biomarker model.*

*As noted by one reviewer, it would be good to enhance some aspects of the reporting depth in relation to the analytical approach/general standards for prognostic studies. Two reporting guidelines may be relevant to refer to here (you may wish to choose whichever seems more appropriate, given your study) - TRIPOD (<https://www.equator-network.org/reporting-guidelines/tripod-statement/>) or STARD (<https://www.equator-network.org/reporting-guidelines/stard/>). If relevant, you can use the checklist for either guideline to inform reporting changes and also append the completed checklist as supporting information when you resubmit the revised paper.

Thank you, we acknowledge the need to conform to reporting standards. Considering this comment and a similar comment, mentioning a different expert consensus paper, from another reviewer, we have adapted the reporting of our results to the TRIPOD recommendations. We have completed and appended the completed checklist as supporting information in the resubmission. To fulfil TRIPOD recommendations (in particular 13b), we have added the text below in the Results and an additional figure in the Supplementary material which was not previously included.

*The distribution of demographics of the validation cohort are very similar to the development cohort(5) (****supplementary material: Table S2****).*

**Reply to Reviewer #1:**

Dear Editors and Authors,
I read article entitled 'High-throughput quantification of fibroblast growth factor 23 and N-terminal pro-B-type natriuretic peptide to identify atrial fibrillation' with great interest.

This original investigation concerns important topic: screening for atrial fibrillation (AF) in patients at high thromboembolic risk.
The abstract incorporates key messages in a concise manner. The general structure of the paper is accurate.

Thank you for your helpful comments.

However, I have some comments to improve the value of the paper:
1.      The predictive ability of the model should be compared with CHARGE-AF risk score and BNP or NT-proBNP.
CHARGE-AF risk score includes only clinical risk factors for AF (age, race, height, weight, smoking status, blood pressure, presence of diabetes mellitus, a history of heart failure, use of antihypertensive medication, and a history of myocardial infarction). Please see:   EP Europace, Volume 16, Issue 10, October 2014, Pages 1426-1433, <https://doi.org/10.1093/europace/euu175>.
This comparison will show whether addition of another biomarker - FGF23 adds significantly to prediction / indication of patients at increased risk of AF.

Thank you. We have included a comparison between the CHARGE-AF model comprising 11 clinical parameters and the present biomarker model in the main manuscript. In our cohort, the biomarker model performed better than CHARGE-AF. We also discussed the uncertainty in determining ‘heart failure’ clinically which renders this factor challenging in screening settings.

The text below was included in the Results:

*The simple CHARGE-AF model (without echocardiography and ECG variables)(8) was calculated for 1057 patients (n = 27 missing data) and compared to the corresponding biomarker model for the same patients. The 5 variable biomarker model yielded an AUC of 0.692 (95%CI 0.661, 0.724) in these patients whereas the 11 variable simple CHARGE-AF model had a lower AUC of 0.637 (95%CI 0.604, 0.670). Discrimination measures for this comparison are presented in the supplementary material (Fig S4).*

The text below was included in the Discussion:

*Existing models such as CHARGE-AF, require a large number of variables to calculate, some of which require specialist input e.g. clinical determination of heart failure. As such, a simple model using age, sex, and BMI and a blood sample for biomarker quantification presents an opportunity to accelerate screening efforts, which in our cohort demonstrated better performance than CHARGE-AF.*

2.      Please propose a nomogram to establish new biomarker-based risk score as e.g. in the ABC (Age, Biomarkers, Clinical history) stroke risk score. Please see e.g. European Heart Journal, Volume 37, Issue 20, 21 May 2016, Pages 1582-1590, <https://doi.org/10.1093/eurheartj/ehw054>

Thank you. We have included the nomogram below in the main manuscript (*Figure 5*).

3.      Figure 3 - NT-proBNP levels were adjusted by age, sex, BMI, and heart failure status. It should be adjusted also at least for renal function. Especially in the light of the statement in conclusions ("Clear elevations of NTproBNP and FGF23 remained after rigorous adjustment for known confounders.") please consider adjustment of the data for more potential confounders. Please see e.g.: Clinical Research in Cardiology (2020) 109:331-338. <https://doi.org/10.1007/s00392-019-01513-y>.

Thank you. Following our predefined statistical analysis plan, we included renal function as a confounder for FGF23. However, we acknowledge that renal function may affect NT-proBNP levels. Therefore, in response to this comment, we have performed additional adjustments (NT-proBNP adjusted for renal function in addition to heart failure [adjustment 2]; FGF23 adjusted for heart failure in additional to renal function [adjustment 4]). We have included these adjustments as a post-hoc sensitivity analysis and added the figure below in the supplementary material (*Figure S2*).

4.      The comparison of models in the same patients - the model calculated using Roche quantified biomarkers (AUC 0.689, 95%CI 0.647, 0.732) and the model using Olink quantified biomarkers (AUC 0.680, 95%CI 0.637, 0.723) should be performed using C-statistic. Moreover, assessment of integrated discrimination improvement (IDI) and net reclassification improvement (NRI) should be done to make firm conclusions.

Thank you. It is our understanding that in a model with a binary outcome such as ours (AF vs no AF), Harrell’s c-statistic/ c-index/ concordance statistic, is equivalent to the area under the receiver operating characteristic curve (AUC) [Harrell et al 1984, *Statistics in Medicine*]. For consistency, we use AUC throughout the manuscript.

We kindly refer the reviewer to the initial report of the current model (Chua et al 2019, reference 5 in the revised manuscript) where the NRI was calculated to quantify the added value of biomarkers. We acknowledge the need to demonstrate discrimination in this validation manuscript and have therefore visualised the discrimination slope for this model and the simple CHARGE-AF model for comparison. We have added the figure below in the supplementary material (*Figure S4*).

5.      In Table S3 data for patients in sinus rhythm should also be provided to make comparison between groups easier.

Thank you, we have added this information in *Table S3*.

6.      Please introduce abbreviations before the first use, e.g. BMI in the abstract or FDA in introduction. The Authors use two abbreviations for N-terminal pro-B-type natriuretic peptide: NT-proBNP and NTproBNP, only the first one should be used.

Thank you, we have corrected these throughout the manuscript.

7.      In the abstract and in the main text - methods section: "≥2 CHA2DS2-VASc stroke risk factors" should be "the CHA2DS2-VASc score of 2 or more' - because as mentioned in another paper originating from the BBC-AF registry (European Heart Journal (2019) 40, 1268-1276) stronger risk factors (age ≥ 75 years old and prior stroke / TIA) could also serve as the only inclusion criteria in the absence of AF.

Thank you, we have corrected these on the abstract and main text.

Abstract: *1625 consecutive patients with either 1) diagnosed AF, or 2) sinus rhythm with CHA_2_DS_2_-VASc score of 2 or more were recruited from a large teaching hospital.*

Methods: *Patients eligible for recruitment either had diagnosed AF confirmed by ECG, or CHA_2_DS_2_-VASc score of 2 or more.*

**Reply to Reviewer #2:**

The paper presents a confirmatory study that extends the authors' previously published work. The present study demonstrates that the published model, which is capable of distinguishing between atrial fibrillation and sinus rhythm, generalizes to an entirely new cohort. As additional validation, the authors show that the presence of AF correlates with the levels of BNP and FGF23 even after correcting for heart failure and chronic kidney disease.

Overall the paper is well-written and utilizes standard statistical tests to assess potential associations between univariate measurements and their ability to detect the presence of AF. In general, confirmatory studies are important, and the presented work provides additional evidence for and confidence in the published model. Unfortunately, no attention is given to the new high-throughput platform mentioned in the paper, which artificially lowers the novelty of the presented work.

Thank you for your helpful comments. We have already briefly described the FGF23 platform in Methods. In response to this comment, we have included the text below in the Methods for more details on the new, high-throughput and near-routine platform in the revised paper.

*…FGF23 was quantified using this pre-commercial assay on a cobas 6000 Elecsys platform (Roche Diagnostics). This in vitro diagnostic (IVD) platform is based on electrochemiluminescence (ECL) technology for heterogeneous immunoassays allowing up to 170 tests/h.*

In the introduction, the authors write "In this study, we developed a novel high-throughput platform for quantifying FGF23, ..." However, the description of this new platform is non-existent. While it is great to see that there is a strong agreement in measurements made with the new and old platforms (Fig 2), it is completely unclear why one would want to use the new platform, if the old one does effectively the same thing. What are some of the features and properties of the new platform (if any) that provide it with an advantage?

Thank you. We have included a more detailed description in the revised manuscript. The key difference is that the new FGF23 assay can be included in routine high throughput machines available in many test centres, while old assays are also accurate, but smaller scale and time consuming even for highly trained research lab staff.

Likewise, the discussion states that "Using a newly developed near-routine, high-throughput, high-sensitivity assay for FGF23 in this validation, we showed that elevated FGF23 in patients with AF is independent of renal function." However, without any description of this novel assay, it is again unclear why the same conclusions couldn't be reached with old methods. The authors should include a brief description of the assay and include a link to a standard protocol repository (e.g., <https://www.protocols.io/>) where more detail is provided.

Thank you, as this is developed as a commercial assay, the protocol is standardised rather than developed in-house. Therefore, it is not deposited on open source platforms. To clarify the advantage of the current high-throughput assays over research assays used previously, we have included the following text in the Discussion:

*In comparison with the research assay platform used in model development, the current high-sensitivity assays validated in this study can be run on routine high-throughput machines already available in many testing labs for immediate, large scale clinical application.*

On a separate note, the paper states that "A two-tailed P value of <0.05 was considered statistically significant." However, given a large number of tests presented in Tables 1 and S2, the authors should include an additional column that shows how p values change after correcting for multiple hypothesis testing.

Thank you. We acknowledge the need for p-value adjustment when multiple testing is performed to reduce type I errors. However, as the baseline tables (Tables 1 and S2) do not involve hypothesis testing and are intended to be descriptive, it is understandable that the p-value is not adjusted and would be read in conjunction with the odds ratio (95%confidence intervals) to appreciate the relationship of the baseline variable with the outcome.

Minor comments:
- Figure 1: define the meaning of orange and blue boxes

Thank you, we have included the text below in the legend of Figure 1.

*Blue boxes indicate the main analysis, orange boxes indicate additional and sensitivity analyses.*

- "The model was fitted with data from the validation cohort..." should read "The model was applied to data from the validation cohort…" (Fitting implies model training.)

Thank you, we have corrected the text in the Results which now reads:

*The model was applied to data from the validation cohort…*

**Reply to Reviewer #3:**

Within the present manuscript Chua et al. aimed to developed a novel high-throughput platform for quantifying FGF23, used a clinically utilized platform for N-terminal pro-B-type natriuretic peptide (NTproBNP), and quantified these biomarkers in the previously tested patients, as well as in an unselected independent cohort of patients presenting to hospital for validation. The authors concluded that routinely available patient characteristics (age, sex, and BMI) combined with elevated NTproBNP and elevated FGF23, quantified on a high-throughput platform, reliably identify patients with AF.
The scientific question is interesting. However, there are several concerns with respect to the design of the study, data analysis, and presentation of results that need to be considered.

Thank you for your comments.

Considering the selection of testes individuals, the authors reported that patients had to present with at CHA2DS2-VASc score of ≥2. It remains unclear why these criteria were applied. The authors need to clarify the rationale behind this definition. The exclusion of low risk (CHA2DS2-VASc score = 0) and intermediate risk (CHA2DS2-VASc score = 1) patients pictures a potentially sicker patient population and does not allow the generalizability of the present results on the general population.
Moreover, it remains unclear why the authors adjusted for different confounders within the multivariate models for both NTproBNP and FGF23 - since both biomarkers have similar potentially confounding variables.

Thank you. The rationale for AF screening is aimed at identifying modifiable risk of stroke and other cardiovascular complications. Patients with none or only one of the CHADSVASc risk factors only have a small risk of stroke, and there is no clear indication for initiation of therapy, (e.g. anticoagulation). Therefore, we focused our development efforts on this group of patients.

We have added the following text to the Introduction:

*As AF screening is aimed at identifying modifiable risks of stroke and other cardiovascular complications, we focused our study on patients with CHA_2_DS_2_-VASc score of 2 or more as there is a clear indication for initiation of therapy (e.g. anticoagulation) in this group of patients.*

Considering the purpose of the present analysis, the discrimination and calibration of the presented data needs to be validated by a - in this regard - more relevant NRI and IDI, which is recommended for prognostic biomarkers and clearly illustrated by Cook (Statistical Evaluation of Prognostic versus Diagnostic Models: Beyond the ROC Curve, Clin Chem. 2008 Jan;54(1):17-23.). Since there is an overwhelming increase of biomarker studies in the field of especially cardiovascular disease with varying quality and questionable clinical utility, the evaluation of biomarkers needs to follow stringent rules and regulations. The current investigation does not fulfill all quality criteria recommended by Ahmad et al. (J Am Coll Cardiol HF 2014;2:477-88.) for the evaluation of a prognostic biomarker in cardiovascular disease, including accurate methodological and statistical procedures (Cox regression models, C-statistic, CART-Analysis, NRI and IDI), gain in prognostic information beyond clinically available information and comparison with other biomarkers. The authors need to adjust their analysis and presentation of the results accordingly in order to comply with recommended quality criteria of biomarker research.

Thank you. We kindly refer the reviewer to the manuscript reporting the initial discovery of the AF prediction model (Chua et al 2019, reference 5 in the current paper, now mentioned in the statistical analysis section), where the NRI was calculated to quantify the added value of biomarkers. As this model was aimed at enriching screening for AF rather than prediction of AF incidence, we have employed a cross-sectional study design and utilised logistic regression (rather than Cox regression) and novel machine learning methodologies, the details of which have been described in the model development manuscript. As we are reporting the validation of this model in this manuscript, we have conformed to the TRIPOD checklist for model validation and have appended the checklist in this revised submission.
