# Supplementary material for: Quantification of fibroblast growth factor 23 and N-terminal pro-B-type natriuretic peptide to identify patients with atrial fibrillation using a high-throughput platform: A validation study
Source: PLoS Med. 2021 Feb 3;18(2):e1003405. doi: 10.1371/journal.pmed.1003405 (PMC7857735; doi:10.1371/journal.pmed.1003405)
Supplement: Attachment — Submitted filename: Revised Production RequestsOct2020.docx [file pmed.1003405.s005.docx]

Revised Production Requests: 

1. Please confirm that you either created the following figure panels, or that you will replace them with an open source alternative: illustrations in your "Summary Figure"

Here are a few open source image websites you may wish to use:
- <https://commons.wikimedia.org>
- <http://www.publicdomainpictures.net/>
- <https://openclipart.org/>

Thank you, we have created the Summary Figure ourselves.

2. Please ensure that all authors have at least one affiliation listed on the title page and that all listed affiliations are attributed to at least one author. Affiliations should be listed in ascending numerical order in the affiliation list and should match what is provided in the Authors Information in the submission form.

Affiliations should include an institution, a city, and a country (no postcode). US affiliations need the state as well, written out in full (with no abbreviations).

Thank you, we think that all our authors fulfil this request. We cannot identify which author/affiliation does not fulfil this request. Please could you provide details if you think there is any remaining issue.

3. Please upload your Supporting Information items individually, rather than in a single file, as you refer to them individually in the text. If you would like to retain the single file please rename internal items (e.g. tables) as A, B, C etc. Please also provide a legend for each under the heading Supporting Information. Supporting Information files should be less than 10 MB each. Please edit any citation of internal items within your Supporting Information files to using letters rather than numbers, e.g. “Table A in S1 Text”. Please ensure these changes are made throughout the manuscript and within the Supporting Information files themselves.

At minimum, your S1 Tables A-E can be one file, but the S1 Figs A-D need to be in a separate file, and legends provided accordingly.

Thank you, we have separated the Tables (S1_supporting_information_Tables) from the Figures (S2_supporting_information_Figures). We added legends to all, we cannot identify any further supporting information item which not have individual legends or which do not follow the naming convention within the main manuscript. Please could you provide details in case you find any remaining issues.

4. Please ensure that each supporting information file has a legend at the end of the manuscript file, after the Reference list.

Thank you. We have added legends for 3 supporting information files at the end of the manuscript file.

5. Please note that your submission cannot contain any references to generic "supporting information" files and instead must refer to specific files using our naming convention (S1 Fig, S1 Table, S1 Text, S1 Data, etc.)

Thank you, we have done so. We cannot identify any references to supporting information which do not refer to specific tables and figures. Please can you provide details if there are still any issues.
